# The Effect of Leaf Litter Cover on Surface Runoff and Soil Erosion in Northern China

**DOI:** 10.1371/journal.pone.0107789

**Published:** 2014-09-18

**Authors:** Xiang Li, Jianzhi Niu, Baoyuan Xie

**Affiliations:** Key Laboratory of Soil and Water Conservation and Desertification Combating of Education Ministry, Beijing Forestry University, Beijing, China; DOE Pacific Northwest National Laboratory, United States of America

## Abstract

The role of leaf litter in hydrological processes and soil erosion of forest ecosystems is poorly understood. A field experiment was conducted under simulated rainfall in runoff plots with a slope of 10%. Two common types of litter in North China (from *Quercus variabilis*, representing broadleaf litter, and *Pinus tabulaeformis*, representing needle leaf litter), four amounts of litter, and five rainfall intensities were tested. Results revealed that the litter reduced runoff and delayed the beginning of runoff, but significantly reduced soil loss (*p*<0.05). Average runoff yield was 29.5% and 31.3% less than bare-soil plot, and for *Q. variabilis* and *P. tabulaeformis*, respectively, and average sediment yield was 85.1% and 79.9% lower. Rainfall intensity significantly affected runoff (*R* = 0.99, *p*<0.05), and the efficiency in runoff reduction by litter decreased considerably. Runoff yield and the runoff coefficient increased dramatically by 72.9 and 5.4 times, respectively. The period of time before runoff appeared decreased approximately 96.7% when rainfall intensity increased from 5.7 to 75.6 mm h^−1^. Broadleaf and needle leaf litter showed similarly relevant effects on runoff and soil erosion control, since no significant differences (*p*≤0.05) were observed in runoff and sediment variables between two litter-covered plots. In contrast, litter mass was probably not a main factor in determining runoff and sediment because a significant correlation was found only with sediment in *Q. variabilis* litter plot. Finally, runoff yield was significantly correlated (*p*<0.05) with sediment yield. These results suggest that the protective role of leaf litter in runoff and erosion processes was crucial, and both rainfall intensity and litter characteristics had an impact on these processes.

## Introduction

Soil erosion has become a serious problem worldwide, causing to decrease productivity of agricultural and forest land, environmental and ecological degradations, and natural disasters such as mudflow that threaten to human safety and infrastructure [1]. Approximately five billion tons of soil is lost annually in China [2], [3]. Soil erosion often damages forest ecosystems, including reduction of soil organic matter content and water-holding capacity [4], loss of valuable soil nutrients, and biota, also declines in biodiversity, and collectively lead to ecosystem instability [1], [3]. While vegetation litter is often considered as an effective cover above soil surface that prevents soil erosion, it is often burned or removed for fuel by local population in many forests of Northern China. These activities may increase detachment of soil aggregates, so raising the sediment generation and transportation via runoff [5].

Although it is widely recognized that vegetation canopy is important in hindering soil erosion [6], by comparison, the role of vegetation litter layers in modulating surface runoff and soil erosion remains poorly understood. Litter layers are known to protect soil from raindrop splashes by intercepting rainfall, preventing surface sealing and crusting of soil, extend the time of soil infiltration, and enhance sediment deposition by increasing soil surface roughness [7]–[9]. Previous studies have mainly evaluated the effectiveness of various surface covers in reducing surface runoff and soil loss, including rock fragments [10]–[13], crop residues [14], [15], grass [16]–[18], geo-textiles [19], post-fire ash and needle cover [20]–[22], and combined cover such as rock and litter [23]. Nonetheless, few leaf litter materials have been tested [24], [25], with variable findings [26]–[34]. The effects of litter layers on surface runoff and soil loss were not consistent across different types of litter, soil, or different environments. In general, runoff volume and sediment yield were reduced in the presence of litter cover [26]–[30]. However, some disputable results have shown that runoff and soil erosion were accelerated by impervious plastic mulch covers [31]–[34].

Moreover, the early studies have mainly examined the percentage of litter cover as a dominant influential factor in surface runoff and soil erosion [20], [23], [28]. Rainfall intensity and litter characteristics such as litter type and areal litter mass (i.e., litter mass per unit area, kg m^−2^), have rarely been regarded as key factors in hydrologic and erosion processes in forest ecosystems. These factors were proved to be effective in rainfall interception [35]–[38], thus possibly exerting strong effects on soil infiltration, surface runoff, and soil erosion.

In this study, the effects of litter cover (i.e., little type and areal litter mass) and rainfall intensity on surface runoff and soil erosion were quantified through rainfall simulation experiments on sloped field plots with the presence or absence of litter covers. The purpose of the present work was to clarify the role of litter layers on surface runoff and soil erosion, which have caused serious environmental and ecological problems and sometimes even catastrophic events such as debris or mudslides in Northern China [39], [40].

## Materials and Methods

### Ethics Statement

The experimental site, Jiu Feng National Forestry Park is managed by the Forestry Committee of Beijing Forestry University and is available for teaching and research of the university. This field study did not involve any endangered or protected species, and the tree species we selected were common in Northern China.

### Study site

**Figure 1:**
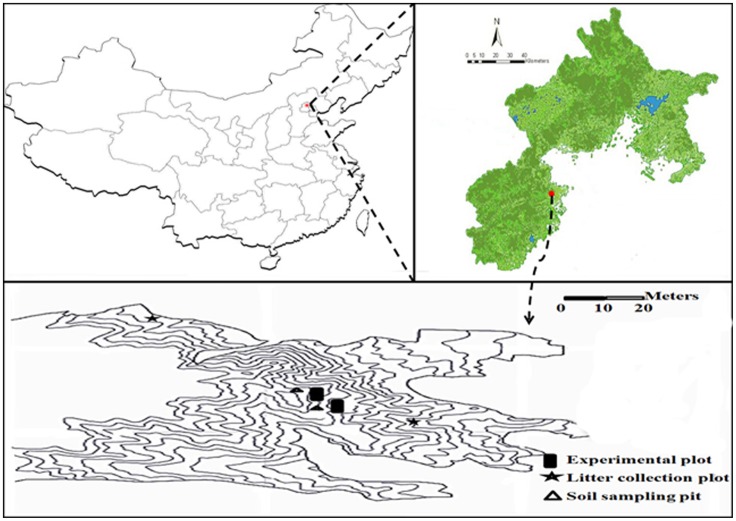
The location of the study area.

This study was performed at Jiu Feng National Forest Park, which is overseen by the Forestry Committee of Beijing Forestry University and available for the university's research and teaching activities. The park is located northwest of Beijing, China (116°28′E, 39°34′N) (Fig. 1). In its warm temperate climate, summers are hot and wet, and winters are cold and dry. Mean daily temperatures are in the range of 23–28°C from May to mid-October, and −5–15°C throughout the winter, resulting in mean annual temperature of 12°C. Mean annual precipitation was approximately 630 mm, most of which occurs as rainfall between June and September, i.e., the period with very intense and erosive rainfall events, usually following the summer drought period. In the park Pinaceae *Pinus tabulaeformis*, Fagaceae *Quercus variabilis*, and Cupressaceae *Platycladus orientalis* are dominant tree species, most of which were planted in the 1950s and 1960s. At an elevation of 70–900 m, soils are mainly consisted of cinnamon soils, changing to brown soils at elevations above 900 m. The average slope is approximately 10% with a northeastern aspect.

### Leaf litter

Collection and characterization of leaf litter used in this study have been described in detail in Li *et al*. [38]. Briefly, because the tree species of *Pinus tabulaeformis* and *Quercus variabilis* are broadly planted not only in the park but also across the Northern China to prevent wind and water erosion, two dominant and typical types of leaf litter was collected and tested: *Q. variabilis* litter (*QVL*), which here represents broadleaf litter, and *P. tabulaeformis* litter (*PTL*), which here represents needle-leaf litter. Regulators and cleaners remove and burn the litter for fuel almost every year. Prior to this study, the most recent removal and burning occurred in April 2010. In order to investigate the natural litter layer distribution, a 25×15 m^2^ experimental stand for each type of litter, respectively. These were further divided into 1×1 m^2^ sub-plots, where human activities were not allowed during the collection period from May 2010 to April 2013. Afterwards, in order to consistently investigate the variation of litter mass to ensure that the measured mass values were representative of field conditions, 10 sub-plots were randomly chosen and litter mass was measured and recorded every four months to cover seasonal variation. A 2-cm-high aluminum rectangular frame was placed over the ground to facilitate observation and collection of fallen leaves in each sub-plot. No cover was set in the sub-plots so that the litter was not isolated from the topsoil, also the biological decomposition of litter was not affected. Because the litter was moved around by wind, the mass of collected litter varied from one sub-plot to another. Because the interface between the litter and soil was clearly distinguishable, the uncomposed litter was selected as the experimental material instead of the fresh leaves, twigs and decomposed litter, only litter with intact shapes were collected. These samples were manually transferred into plastic bags, and transported to the laboratory, where they were allowed to air-dry.

The lengths and widths of the collected needle-leaf litter and broadleaf litter were measured (Table 1). Because the twigs and needles were difficult to separate, they were weighed together, and decomposed needles and broadleaves were excluded from the measurements. The areal litter mass in kg per m^2^ was then calculated by dividing the weighed litter mass over the sub-plot areas. The undecomposed litter was chosen for two main reasons: first, it was the dominant component of the litter (approximately 85%) and was easy to identify; second, the undecomposed litter was located in the upper layer where it would directly intercept the raindrops. For this reason, that the undecomposed litter was assumed to play a more significant role in modulating surface runoff and soil erosion processes than decomposed litter and half-decomposed litter. Accordingly, the mass of this component of litter ranged from 0.33 to 1.24 kg m^−2^ for *QVL* and from 0.18 to 0.77 kg m^−2^ for *PTL* in the sub-plots (1×1 m^2^). Based on the measurements mentioned above, to represent the litter variation characteristics and erosion in the slope accurately and to effectively illustrate and compare the hydrological and erosive responses of the two types of litter, four areal litter masses of 0.3, 0.5, 0.8, and 1.0 kg m^−2^ were selected for testing in rainfall simulation studies. This may explain the role of litter in runoff and sediment depletion.

**Table 1:** Forest stands and litter characteristics [38].

Litter species	Plot area (m^2^)	Density (trees ha^−1^)	DBH* (cm)	Height (m)	Leaf litter length (cm)	Leaf litter width (cm)
*Q. variabilis*	25×15	1225	11.2	9.8	11.3–13.7	3.5–4.3
*P. tabulaeformis*	25×15	1748	7.6	5.2	9.5–12.5	3.3–5.1 (twigs + needles)

*Diameter at breast height.

### Experimental plot design

**Figure 2:**
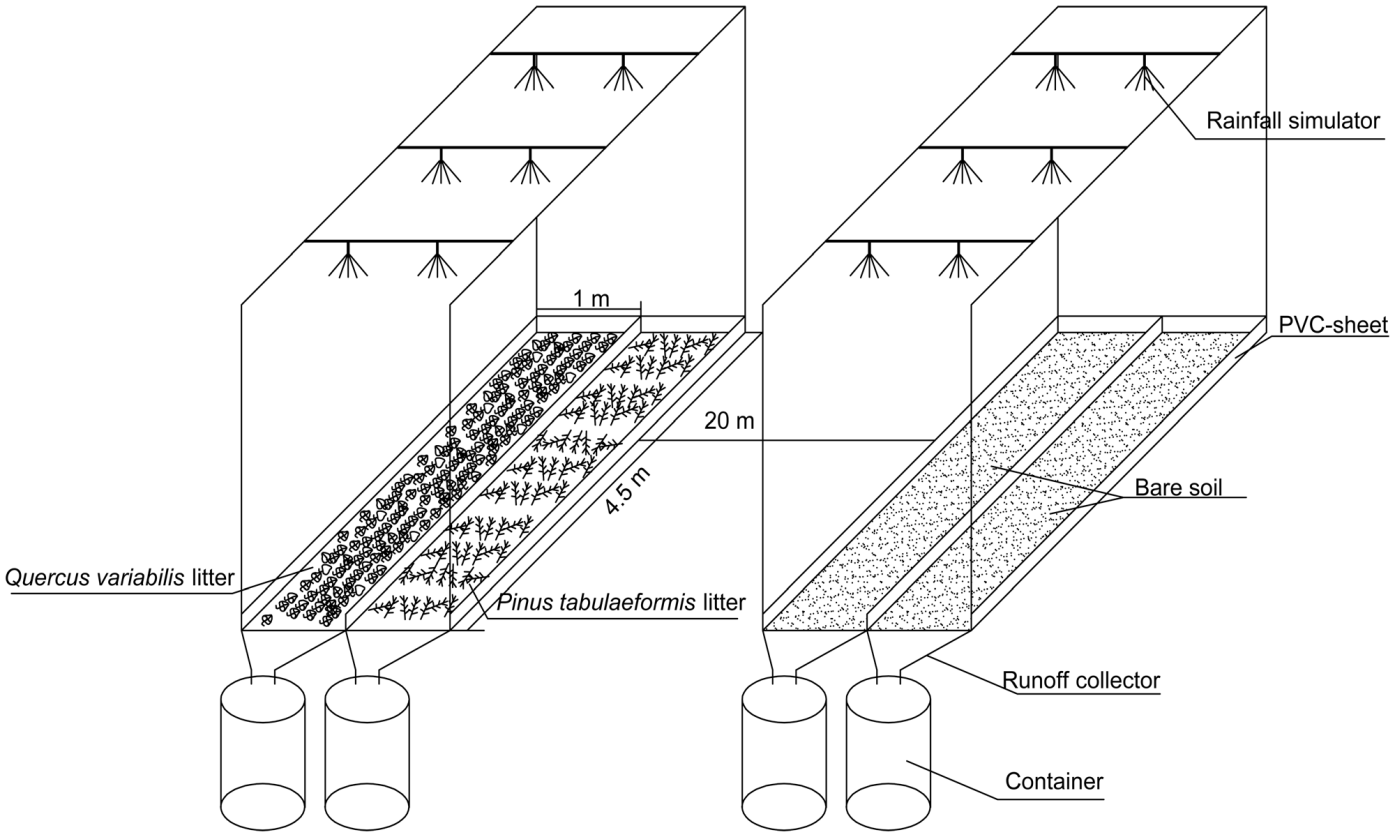
A single set of schematic diagram of experimental runoff plots.

Based on initial site survey, two northeastern-facing open runoff plots (4.5×2 m^2^ each) with an approximate slope of 10% were selected for rainfall simulation experiments. These served as the bare plot and the litter-covered plot, respectively. The plots were approximately 20 m apart, the soil moisture and soil texture composition were almost the same, so they were not decisive factors in the infiltration and runoff generation in further analysis of runoff yields. All large stones were removed from both plots, and the remaining decomposed litter was removed from the litter-covered plot. No trees or other shrubs were present in either plot. Both plots were divided in half with each part measuring 4.5×1 m^2^. The two halves of the divided plots were then covered with *Q. variabilis* litter (*QVL*) and *P. tabulaeformis* litter (*PTL*). These served as litter-covered plots in comparisons of the effects of the two different types of litter on runoff and erosion control (Fig. 2). Before each test run, leaf litter was brought out from plastic bags, then carefully distributed by hand in the litter-covered plot at litter masses of 0.3, 0.5, 0.8, and 1.0 kg m^−2^. Litter thickness was measured in the upper, middle, and lower part of the plot to test whether the litter covered soil surface uniformly. Several adjustments were made if the litter thickness varied by more than 0.5 cm. The remaining two plots (4.5×1 m^2^ each) were regarded as the control (or bare-soil) plot with no litter cover. Within almost 30 min of the cessation of rainfall, leaf litter was re-collected in plastic bags and then oven-dried at 80°C, and evenly returned to the plots before the next rainfall simulation.

### Soil properties

The soil properties of each plot are presented in Table 2 (A common Chinese Soil Taxonomy was reported by CRGCST [41] and Shi *et al*. [42]). To comprehensively represent the soil characteristics of the experimental plot, bulk soil samples were collected (at a depth of 0–40 cm) from three different points outside each plot (2–5 m away from the plot) to determine soil properties from May to June, 2013. At each point, soil samples were separated into four sub-samples based on soil depth of 10 cm (0–10 cm, 10–20 cm, 20–30 cm, 30–40 cm) and stored in aluminum specimen boxes and trays. In order to determine soil bulk density (*D_b_*) a weight basis of a 100 cm^3^ soil core sample was collected under field-moisture conditions, oven-dried at 105°C for 24 h, and measured for the mass of oven-dried solids, *V*_t_ refer to the bulk volume of the soil, which includes the volume of the soil (*m*_s_). Then *D_b_* was calculated as *m*_s_/*V*_t_ and the pore space between the soil particles [43], here *V*_t_ = 100 cm^3^. Total porosity *S*_t_ can be calculated from the particle density (*D*_p_) and bulk density (*D*_b_) as follows:
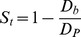



**Table 2:** Soil properties (0–40 cm deep) in experimental and control plots.

	Bare-soil plot	Litter-covered plot
Soil moisture (%)	21.2	17.5
Porosity (%)	57.4	55.4
Bulk density (g cm^−3^)	1.57	1.43
pH	5.6	5.7
Sand (%)	79.5	73.6
Silt (%)	10.9	12.5
Clay (%)	9.6	13.9

Here, particle density (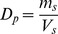) refers to the mass (*m*_s_) of a unit volume of solid soil particles (*V*_s_) [44]. Gravimetric soil water content (water mass/dry soil mass) was also measured after the samples were oven dried [45]. Soil particle size distribution was measured after the soil samples were treated with H_2_O_2_ and dispersed in sodium hexametaphosphate solution using the Bouyoucos densimeter method [46], [47]. Soil pH was determined with an electrode pH-meter on the saturated soil paste of 1∶2.5 soil to distilled water ratio [48], [49].

### Rainfall simulation

The relatively high variability of the rainfall intensity in Northern China makes it difficult to examine its role in rainfall-runoff and soil erosion processes that take place under litter cover [50], [51]. Here, simulated rainfall produced by an artificial rainfall simulator jointly developed by Beijing Normal University and Beijing Jiaotong University in 2006 was used [52], [53]. A detailed description on the rainfall simulator was provided in Li *et al* [38]. The simulator delivered water at a height of 4.5 m and at varying speeds, spraying an area 2.2 m long ×1.5 m wide under a range of rainfall intensities. This rainfall simulator was designed to allow raindrops to reach terminal velocity and allow the flow rates to be easily controlled, resulting in simulated stable rainfall events with 80% uniformity and raindrop sizes of 2.3±0.3 mm. Simulated rainfall chemistry is also an important factor to be considered in the simulation experiments. Water with high electrical conductivity tends to flocculate soil particles, and water with low electrical conductivity typical of natural rainfall may disperse and readily erode the same soil particles [54], [55]. Distilled water was modified using NH_4_Cl and NaCl to produce a solution with a pH of 5.5 and electrical conductivity ranged from 12.2 to 20.7 µS cm^−1^ (a detailed description of the electrical conductivity measurement was presented in the National Standard of China [56]), which was similar to the reference value of 14.8 µS cm^−1^ [57].

According to the precipitation data in the study region over 53 years from 1956 to 2008, the maximum rainfall intensity between June and September ranged from 2.6 to 82 mm h^−1^ [58], each plot was subjected to rainfall at intensities of 5.7, 11.7, 25.2, 49.8, and 75.6 mm h^−1^ for 1 h each to represent natural rainfall conditions and to measure the hydrological and erosive response to rainfall accurately. The duration of simulated rainfall was 1 h, which was consistent with the duration of the stable natural rainfall in the study region since 1984 [59], [60]. Runoff and sediment samples were collected using a metal runoff collector, which was placed at the bottom of each plot to capture the runoff during the test run at 1-minute intervals, and the runoff sample was weighed using an electronic balance. Sediment was settled, separated from the water, and oven-dried at 105°C for 24 h, after which it was weighed to determine the sediment yield. Time to runoff (*T_r_*) was measured as the time when runoff started to develop on the plot surface after the rainfall began [21], [61]. Within 30 min after the cessation of rainfall, by which the gravitational water had drained out of the litter, the litter was removed from the soil manually and collected in two plastic bags.

Runoff coefficient (*R_c_*) defined as the proportion of total rainfall that becomes runoff during a storm event, is used to describe the variation of runoff and water resource development [62]. *R_c_* was calculated for each rainfall event. Sediment concentration (*SC*) was determined by the equation 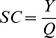, where *Y (g)* represents sediment yield, and *Q (L)* represents runoff volume. In addition, infiltration and the antecedent soil water content affected runoff process very visibly [63]. Given the wide range of simulated rainfall intensities, the simulated rainfall intensity (*RI*) order 5.7, 25.2, 49.8, 11.7, and 75.6 mm h^−1^ was set to avoid that two high-intensity of rainfalls were simulated consecutively. To lessen the impact of infiltration on runoff and sediment generation, changes in soil water content in the soil profiles were measured by inserting time-domain reflectometry probes to a depth of 20 cm before and after the experiment at 2-day (48-h) intervals to determine whether the soil water content had reached 20%±2% (Table 2). If the soil water content reached or exceeded 25%, the rainfall simulation would be delayed for another 24 h until the initial water content before every experimental run was 20%±2%. All test runs were carried out from August to September 2013, during which no intensive rain or significant wind occurred. Each test was repeated once.

### Statistical analysis

Linear, polynomial and non-linear regressions were used to determine the relationship between the litter mass (or *RI*) and runoff parameters (or sediment parameters). Correlation analysis among runoff (e.g. runoff yield, *R_c_*, *T_r_*), and sediment (e.g. sediment yield, *SC*) as dependent variables and litter mass and of *RI* as an independent variable was performed to evaluate possible relationships among them, and to facilitate understanding of the hydrological and erosion processes. Parameters were considered to be significantly correlated when they were at or above the 95% confidence level (*p*≤0.05). A test of normality was conducted by comparing the Sig. value in Kolmogorov-Smirnov and Shapiro-Wilk. Homoscedasticity was carried out to determine the whether the data was homogeneous. Sig>0.05 was defined as homoscedasticity in the Levene Statistic. One-way analysis of variance (ANOVA) was carried out to determine if there were any differences in runoff and sediment yield among three plot treatments (one bare-soil, two litter-covered plots), specifically differences between the broadleaf litter-covered plot and needle-leaf litter covered plot. The Fisher LSD (Least Significant Difference) test at *p*≤0.05 was used to test for significant differences. Data were grouped by treatment (bare, *Q. variabilis* litter cover and *P. tabulaeformis* litter cover). All statistical analyses were performed using IBM SPSS Statistics 20.0 software.

## Results and Discussion

### Runoff

Runoff yield and runoff coefficient (*R*_c_) data are presented in Table 3. Generally, the most runoff took place in the control plot (bare soil). The mean amount of runoff in the *QVL* and *PTL* cover plots was lowered by 29.5% and 31.3% compared to the bare plot, respectively. In agreement, similar results were reported by Singer and Blackard [28], Pannkuk and Robichaud [20], Findeling *et al*. [15], and Cerdà and Doerr [21], with a reduction that ranged from 0.12–55.6% in contrast with the bare soil. This was likely due to the protection provided by the litter cover, which absorbed the energy of raindrops and also increased the roughness of soil surface to increase infiltration rate, and delayed and reduced runoff [49]. However, the opposite result was observed for straw mulch cover: runoff yield was slightly higher (by 0.09%) than that in a bare-soil plot [28], [33]. Similar results were obtained for rock fragments cover [12], [13], [64] and plastic mulch cover [34], presumably because the physical shapes or impervious nature of these surface covers concentrated the flow of water to decrease the infiltration rate and increase the flow velocity [25], [65]. However, results of LSD test in ANOVA revealed no significant differences (*p*≤0.05) in runoff yield between the control and two litter-covered plots (*p* = 0.417 and 0.390 respectively). In contrast, significant differences (*p*≤0.05) were found in *R*_c_ (*p* = 0.000 and 0.001) and *T_r_* (*p* = 0.028 and 0.012) between bare and litter-covered plots. The results implied that litter cover had an impact on runoff, but other factors, such as precipitation and rainfall intensity should be taken into account as well.

**Table 3:** Runoff yield and runoff coefficient data corresponding to each treatment and simulated rainfall event.

Type of cover	Litter mass (kg m^−2^)	Rainfall intensity (mm h^−1^)				
		5.8	11.8	25.2	49.8	75.6
		Runoff yield (mm), runoff coefficient (%)				
Bare soil	0	2.09, 36.7	6.58, 56.2	21.3, 84.5	43.98, 88.3	68.42, 90.5
*Q. variabilis*	0.3	0.81, 14.2	4.64, 39.7	13.64, 54.1	26.71, 53.6	51.05, 67.5
	0.5	1.28, 22.5	3.66, 31.3	13.24, 52.5	26.53, 53.3	54.50, 72.1
	0.8	0.98, 17.2	3.66, 31.3	13.43, 53.3	33.26, 66.8	52.10, 68.9
	1.0	0.28, 4.9	2.75, 23.5	13.79, 54.7	33.14, 66.6	51.90, 68.7
*P. tabulaeformis*	0.3	0.71, 12.5	2.98, 25.5	12.31, 48.9	35.74, 71.8	52.06, 68.9
	0.5	0.65, 11.4	3.06, 26.2	11.58, 46.0	29.10, 58.4	43.54, 57.8
	0.8	0.69, 12.1	3.29, 28.1	9.60, 38.1	28.82, 57.9	54.24, 71.8
	1.0	0.35, 6.1	3.16, 27.0	12.76, 50.6	36.35, 73.0	50.30, 66.5

The manner in which litter type (broadleaf *vs* needle-leaf), specially differences in physical leaf shape, might affect runoff was studied. However, no significant differences in runoff yield (*p* = 0.696), *R_c_* (*p* = 0.677) and *T_r_* (*p* = 0.681) were found between *QVL* and *PTL* plots, indicating that litter type was a the major influencing factor in controlling runoff in the present study as it was supposed to be. Very few studies concentrated on the role of litter type in runoff reduction, particularly on the physical differences in leaf shape. In a previous work, Neris *et al*. argued that runoff from the pine needle litter cover was twice of the rainforest litter cover, which does not match the current results [47]. The discrepancy suggests that further research is required.

**Figure 3:**
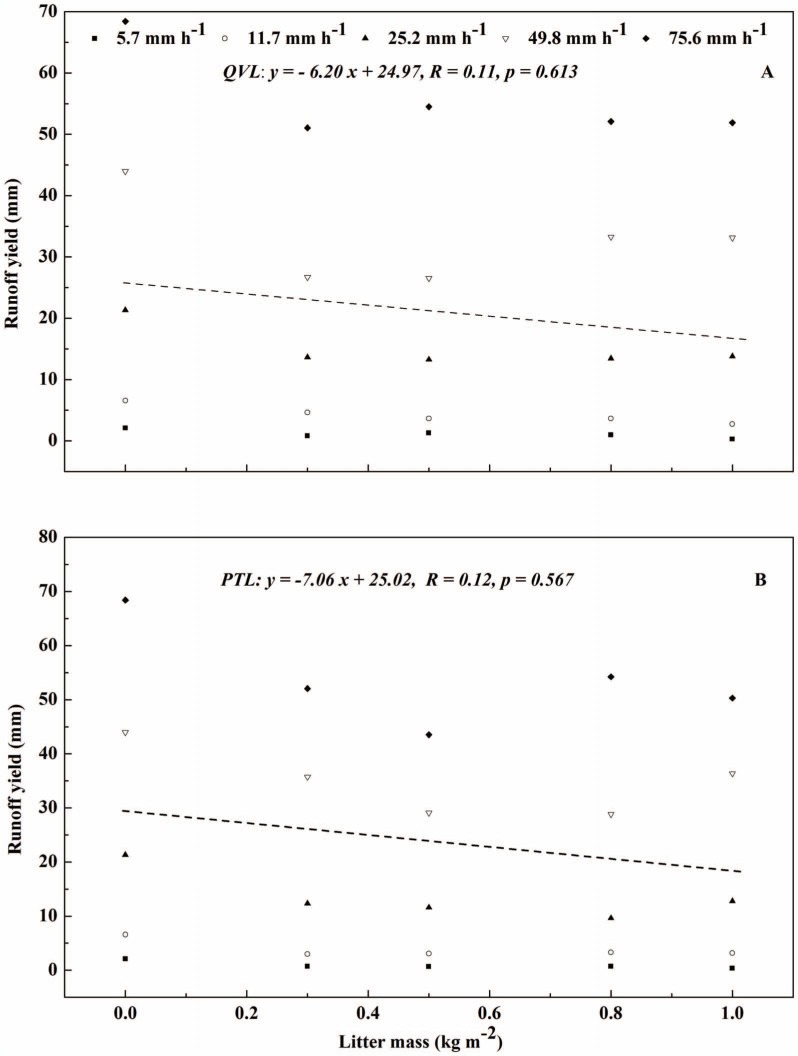
Runoff yield versus litter mass for five rainfall intensities for bare-soil plots (Litter mass equals zero represents ‘bare’), *Quercus variabilis* litter (A), and *Pinus tabulaeformis* litter (B).

**Figure 4:**
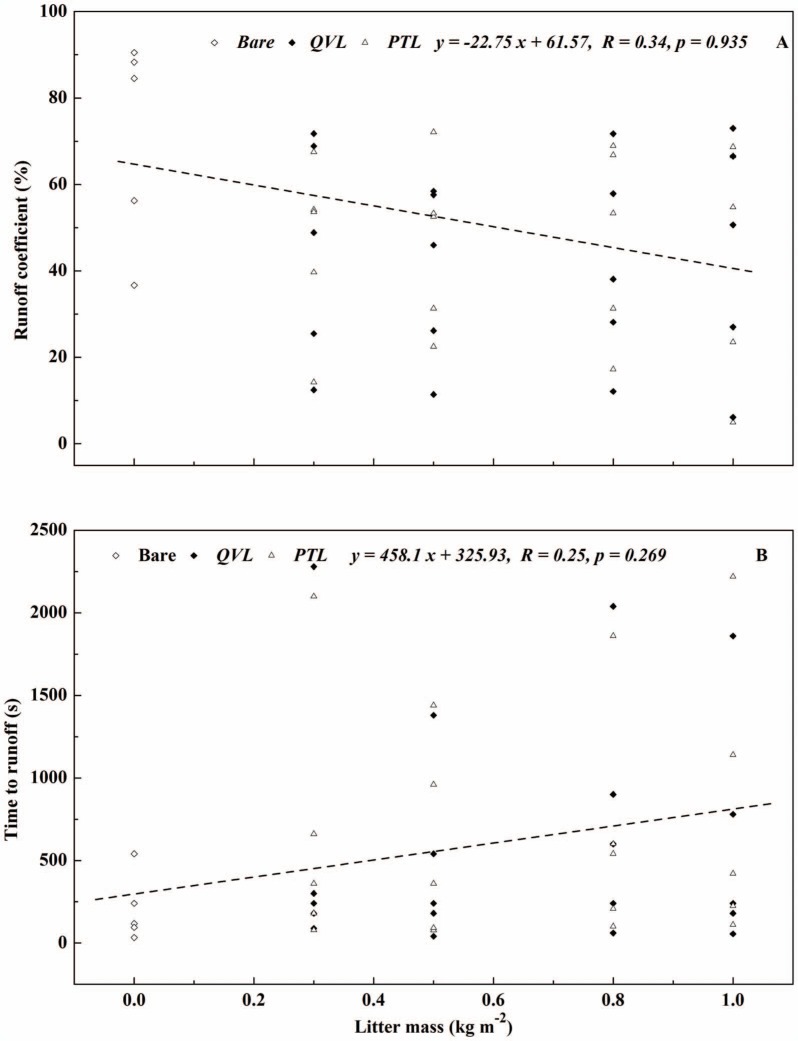
Runoff coefficient and time to runoff versus litter mass for bare-soil plots (open diamonds), *Quercus variabilis* litter (closed diamonds), and *Pinus tabulaeformis* litter (open triangles) for five rainfall intensities.

Litter mass was treated as an influencing element in runoff process by covering the topsoil, but its effect on runoff remains unclear. In general, no significant linear correlations were found between litter mass and runoff yield for *QVL* (*R* = 0.11, *p* = 0.923) and *PTL* (*R* = 0.12, *p* = 0.964) (Fig. 3), and no non-linear correlations were observed. Runoff yield decreased dramatically when litter mass increased from 0 to 0.3 kg m^−2^ but then remained steady regardless of the increasing litter mass afterwards, which proved that litter cover had a strong impact on runoff reduction as stated above. *R*_c_ (*R* = 0.34, *p* = 0.114) and *T_r_* (*R* = 0.25, *p* = 0.078) showed a similar correlations with litter mass (Fig. 4). One likely explanation was that the increased litter mass might intercept and store more rainwater. However, on the other hand, it may provide a flow channel for runoff by covering more bare topsoil so that runoff flowed on rather than through the leaf litter, particularly under rainfall intensities of 49.8 and 75.6 mm h^−1^. For this reason, more runoff was observed with higher litter masses of 0.8 and 1.0 kg m^−2^. While Findeling *et al*. compared the efficiency of different corn residue masses (0, 0.15 and 0.45 kg m^−2^) in cutting runoff down, and reported that mean values of *R_c_* were 0.44, 0.16, and 0.05, respectively [15]. They confirmed that surface cover contributed notably to the decrease of runoff in contrast with the bare soil, but because they tested only a narrow range of masses,, the slight increase in mass was not sufficient to facilitate extrapolation that runoff would decrease as the ground cover mass increased, which suggested that a broader range of mass should be studied in further works.

**Figure 5:**
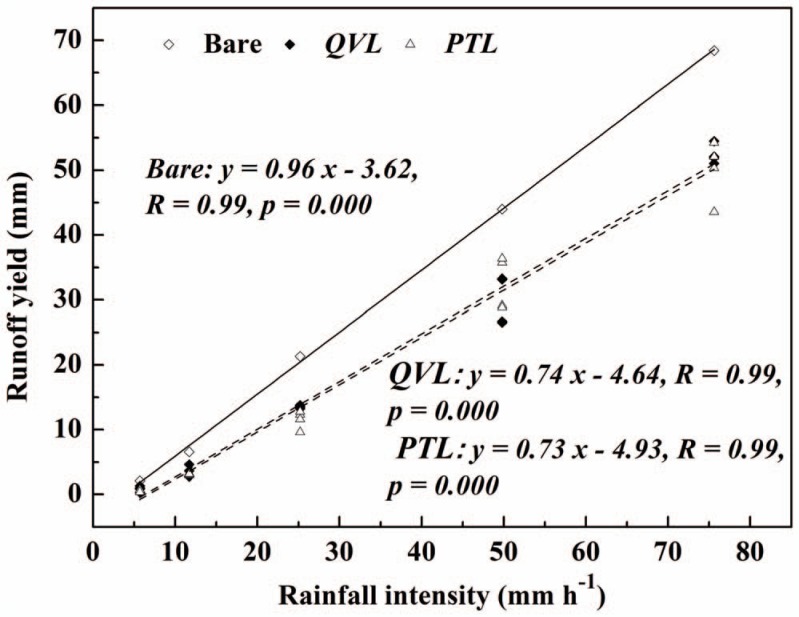
Runoff yield versus rainfall intensity for bare-soil plots (open diamonds), *Quercus variabilis* litter (closed diamonds), and *Pinus tabulaeformis* litter (open triangles) for four litter masses.

**Figure 6:**
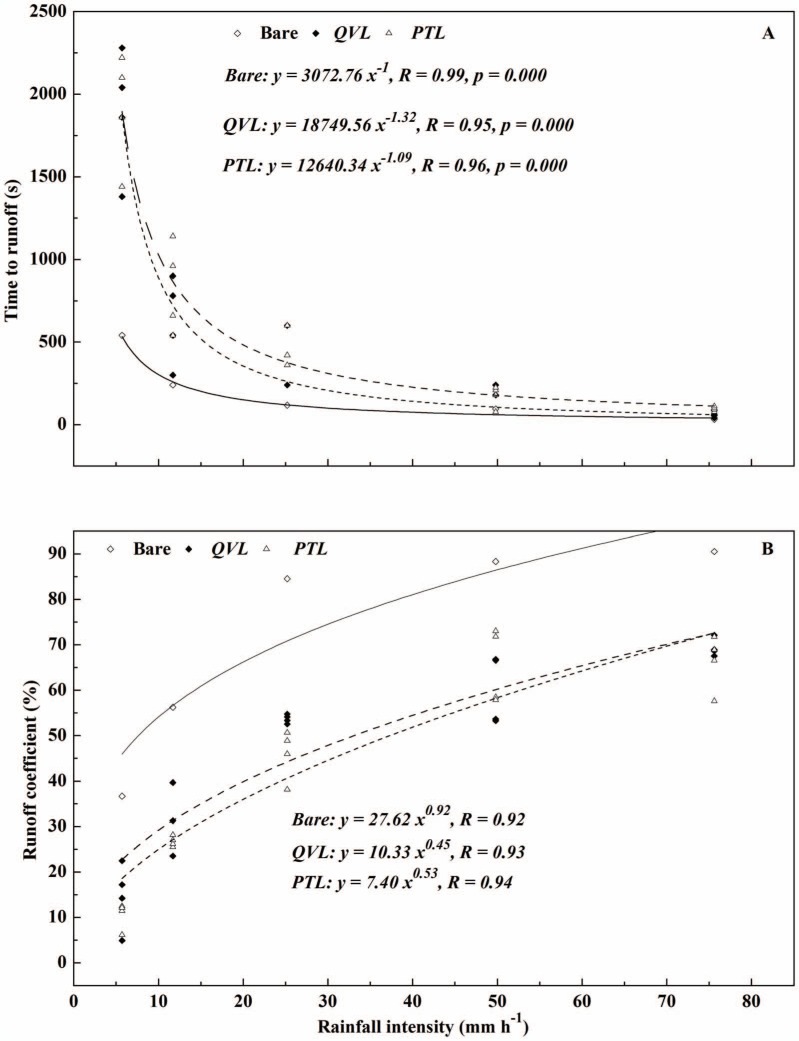
Rainfall intensity versus time to runoff and runoff coefficient for bare-soil plots (open diamonds), *Quercus variabilis* litter (closed diamonds), and *Pinus tabulaeformis* litter (open triangles) for four litter masses.

Significant linear correlations were found between rainfall intensity (*RI*) and runoff yield for all the plots in Figure 5 (*R* = 0.99, *p*<0.05). In all plots, runoff yield increased with increasing *RI*. Runoff yield at the maximum *RI* of 75.6 mm h^−1^ was 32.7, 62.4, and 83.4 times larger than that at the minimum *RI* of 5.7 mm h^−1^ in bare, *QVL* and *PTL* plots respectively. Based on regression analysis, *R_c_* and time to runoff (*T_r_*) showed a significant power (*R*>0.9, *p*<0.05) and reverse-power (*R*>0.95, *p*<0.05) relationships with *RI* (Figure 6A and 6B). As expected, mean *R_c_* increased from 12.6% (*RI* = 5.7 mm h^−1^) to 67.8% (*RI* = 75.6 mm h^−1^) in litter-covered plots (Figure 6B), but *T_r_* decreased apparently from nearly 30 min to 1∼1.5 min with increasing *RI*. The findings were in accordance with those reported by Gholami *et al*. [65], who noted that mulch cover was more effective in controlling runoff at a low *RI* of 30 mm h^−1^ (*R_c_* = 49.9%, *T_r_ = *81.83 s) than at a high *RI* of 90 mm h^−1^ (*R_c_* = 66.6%, *T_r_* = 55.22 s). The phenomena in the present study also proved that *RI* (rather than litter mass) was a dominant factor in determining runoff irrespective of litter cover. This was probably because when *RI* was high, leaf litter was pressed close to the soil surface, blocking the channels between leaves. The accumulative precipitation was assumed to surpass infiltration rate, which resulted in the generation of more runoff.

### Sediment

The magnitude of the sediment yield and sediment concentration (*SC*) are shown in Table 4. Generally, bare-soil plot generated 2.75 kg sediment, which was almost 6.7 and 5 times larger than that in *QVL* (0.41 kg) and *PTL* (0.55 kg) plots, respectively. Further, ANOVA analysis indicated there were significant differences (*p* = 0.000 and *p* = 0.001) between bare plot and two litter-covered plots. Similar results were obtained in terms of *SC* in bare and litter-covered plots (*p* = 0.001 and 0.003). In this way, litter cover was able to retard erosion. The observed outcomes were similar to those presented in previous studies. For example, Pannkuk and Robichaud reported that rill and inter-rill erosion were reduced by 70% when plots were covered with Douglas fir needles and 30% when they were covered with Ponderosa pine needles, as compared with the erosion in a bare-soil plot [20]. Cerdà and Doerr reached similar conclusions in their study on soil erosion from the plots covered with ash and pine needles [21]. Benkobi *et al*. suggested that soil loss was reduced by about 87% in 100% litter cover relative to bare soil [23]. Miyata *et al*. reported that annual soil erosion in uncovered plots was 3.7 times greater than in covered plots [30]. However, Singer and Blackard found that more soil loss was observed when mulch covers <40% in contrast with bare soil [28], this was mainly because they excluded the splash erosion off the inter-rill plot, and overall sediment was reduced under mulch cover.

**Table 4:** Sediment yield and sediment concentration data corresponding to each treatment and simulated rainfall event.

Type of cover	Litter mass (kg m^−2^)	Rainfall intensity (mm h^−1^)				
		5.8	11.8	25.2	49.8	75.6
		Sediment yield (kg), sediment concentration (g L^−1^)				
Bare soil	0	0.17, 18.29	0.23, 7.77	1.65, 17.26	4.70, 23.73	7.02, 22.79
*Q. variabilis*	0.3	0.07, 17.89	0.13, 6.19	0.79, 12.91	0.58, 4.79	2.30, 10.02
	0.5	0.07, 12.00	0.14, 8.42	0.51, 8.53	0.55, 4.64	1.24, 5.06
	0.8	0.05, 10.88	0.08, 4.98	0.18, 2.96	0.31, 2.09	0.42, 1.80
	1.0	0.06, 49.37	0.14, 11.39	0.16, 2.63	0.16, 1.10	0.22, 0.96
*P. tabulaeformis*	0.3	0.10, 30.14	0.12, 8.61	0.21, 3.77	0.85, 5.29	3.38, 14.44
	0.5	0.06, 19.97	0.15, 10.65	0.32, 6.14	0.68, 5.21	2.94, 15.03
	0.8	0.06, 17.97	0.09, 5.89	0.17, 3.92	0.50, 3.86	0.52, 2.11
	1.0	0.04, 24.44	0.13, 9.17	0.18, 3.08	0.26, 1.58	0.32, 1.40

In the present study, because the litter cover prevented raindrops from hitting the soil surface directly by intercepting and storing the rainfall, the detachment of soil aggregates and splash erosion was lessened [66], [67]. Less soil was lost in the litter-covered plots than in the bare plot.

Additionally, mean sediment yield and *SC* in the *QVL* plot were slightly lower than in the *PTL* plot, but no statistical differences were found in sediment yield (*p* = 0.544) and *SC* (*p* = 0.810) between the two plots. The findings indicated that litter type (broadleaf or needle-leaf) was may not be a defining predictor in reducing soil loss. This was probably because even though more topsoil was exposed and soil aggregations were detached under needle-leaf litter cover compared with broadleaf litter, needle-leaf litter tended to form mini-debris dams to trap down the soil particles in the runoff and return them to the soil surface [20]. The results were consistent with Neris *et al*., who also reported that no significant differences were observed between pine forest floor (including needle leaf litter) and a rainforest floor (including broadleaf litter) [47].

**Figure 7:**
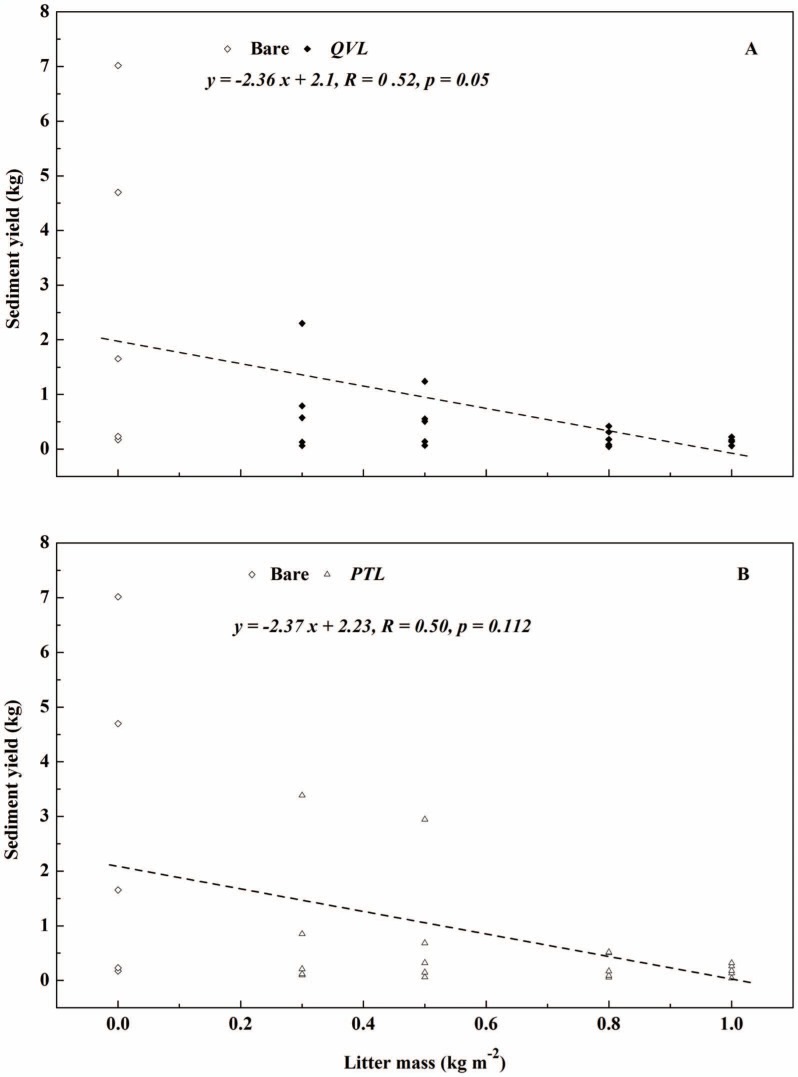
Relationship between litter mass and sediment yield for bare-soil plots (open diamonds), *Quercus variabilis* litter (closed diamonds), and *Pinus tabulaeformis* litter (open triangles) for five rainfall intensities.

**Figure 8:**
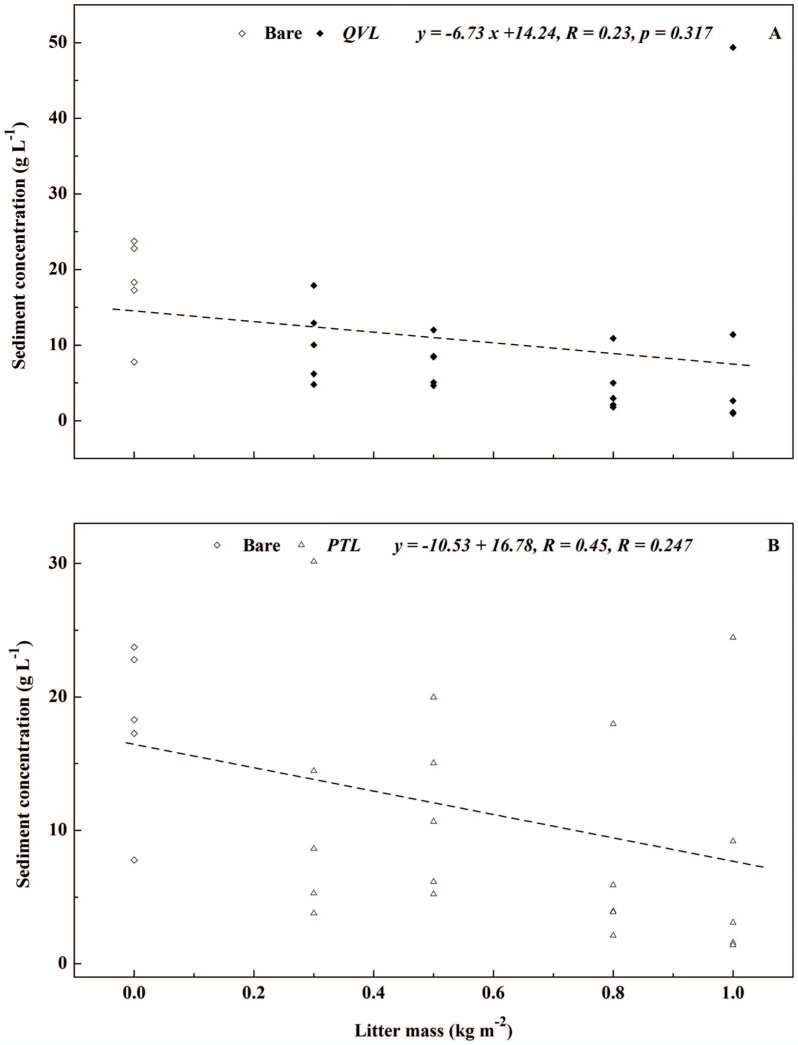
Relationship between litter mass and sediment concentration for bare-soil plots (open diamonds), *Quercus variabilis* litter (closed diamonds), and *Pinus tabulaeformis* litter (open triangles) for four litter masses.

Linear relationships between litter mass and sediment yield in *QVL* (*R* = 0.50, *p* = 0.05) and *PTL* plot (*R* = 0.52, *p* = 0.112) are shown in Figure 7. In general, total sediment yield decreased from 13.77 to 0.75 kg (*QVL*) and 0.92 kg (*PTL*) when the litter mass increased from 0 (bare) to 1 kg m^−2^, but the largest decreases of 71.9% and 66.2% were revealed when litter mass increased from 0 to 0.3 kg m^−2^. Similar relationships are presented in Figure 8 in terms of litter mass and *SC*. *SC* decreased as litter mass increased. Although no significant correlations were observed, the effect of litter mass on reducing sediment was found to be relevant, as the increasing litter mass covered the remaining bare soil, which indirectly protected soil from raindrop impact, and reduced the velocity of runoff [15], [67]. Similarly, Lal reported that soil loss decreased from 52.4 to 0.04 kg m^−2^ when the maize grain yields increased from 0.06 to 0.68 kg m^−2^ [68].

**Figure 9:**
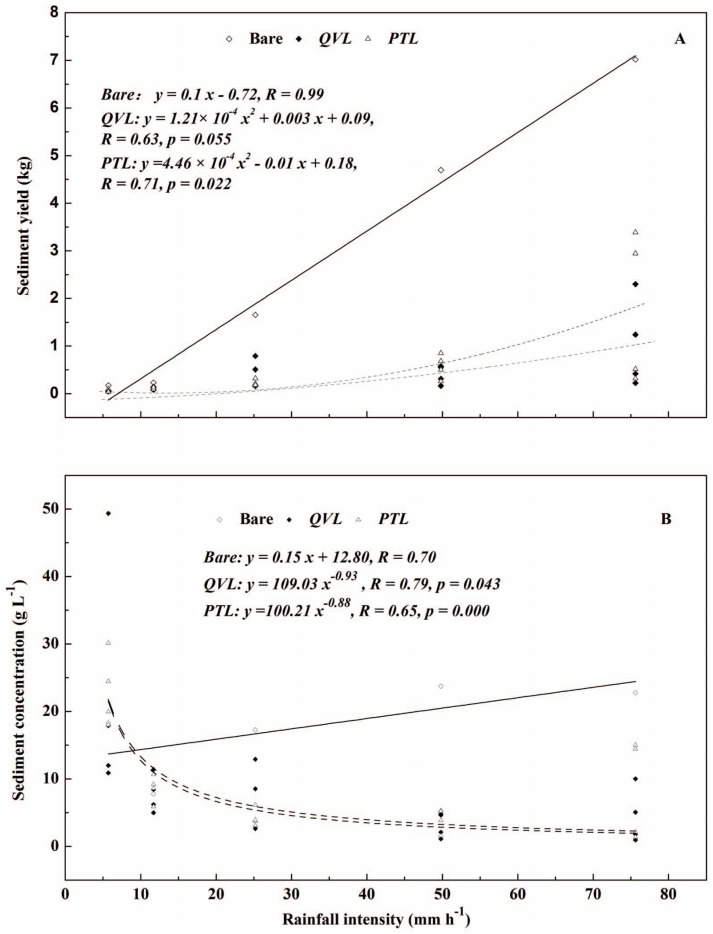
Relationships between sediment yield (A) and sediment concentration (B) versus rainfall intensity for bare-soil plots (open diamonds), *Quercus variabilis* litter (closed diamonds), and *Pinus tabulaeformis* litter (open triangles) for four litter masses.

*RI* also had an impact on sediment generation. Both linear (bare soil: *R* = 0.99) and polynomial relationships (*QVL*: *R* = 0.63, *p* = 0.055; *PTL*: *R* = 0.71, *p* = 0.022) between *RI* and sediment yield are presented in Figure 9A. When *RI* increased from 5.7 to 75.6 mm h^−1^, sediment yield increased 41.3, 17.5, and 29.8 times in bare, *QVL* and *PTL* plots, respectively. *SC* showed a reverse-power decrease trend with *RI* in *QVL* and *PTL* but increased in bare plots. These results verified the protective role of the leaf litter layer, which was in line with the previous studies [24], [25], [46]. This was mainly because on one hand, leaf litter reduced the detachment of soil aggregation by covering on the topsoil, particularly, high-intensity rainfall (49.8 and 75.6 mm h^−1^) with intense striking force pressed leaf litter closer to the soil surface [17]; on the other hand, the coverage by leaf litter increased the surface roughness, which facilitated sediment deposition in the runoff [30], [67].

### Relationship between runoff and sediment

**Figure 10:**
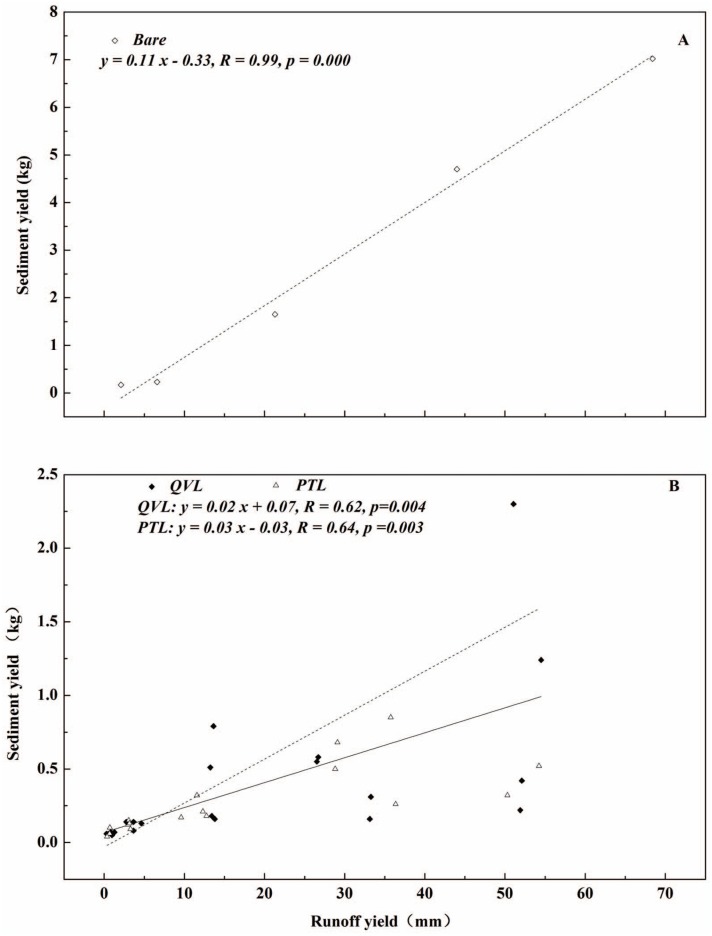
Relationships between runoff yield and sediment yield for bare-soil plots (open diamonds), *Quercus variabilis* litter (closed diamonds), and *Pinus tabulaeformis* litter (open triangles).

As analyzed above, runoff yield in the litter-covered plots was not significantly different (*p* = 0.226 and 0.220) from that in the bare plot, but significant differences (*p* = 0.000 and 0.000) were shown in sediment between the bare plot and litter-covered plots. This can be explained briefly by the different forms of interaction between litter type and runoff-erosion process. For broadleaf litter, runoff generated and flowed on the leaf surface, while the presence of needle-leaf litter increased the soil surface roughness and trapped down soil particles in the flow as a barrier [15], [21], [67], [69]. Significant correlations (*p*≤0.05) between runoff and sediment were observed for plots in Figure 10A and 10B. Apparently, sediment yield increased with the increasing runoff. This is similar with the previous studies (e.g. [30], [65], [70]). In addition, an excellent linear regression was observed in bare plot (*R* = 0.99). The greatest runoff, which showed large amounts of energy, was capable of detaching soil aggregation and moving the soil particles. However, leaf litter reduced runoff and sediment by covering soil to prevent splash erosion and largely increasing surface roughness which in turn increased infiltration. The phenomenon confirmed again that litter cover played an irreplaceable role in runoff and soil erosion control.

## Conclusion

The research effort was conducted to examine the role of leaf litter in runoff and soil erosion. Results showed that runoff and sediment yield were lower in litter-covered plots than in bare plot, litter cover had a relevant impact on runoff and sediment control. A detailed analysis of how runoff and sediment interacted with litter type, litter mass and rainfall intensity showed that rainfall intensity rather than litter type (broadleaf or needle-leaf) or litter mass was the decisive factor in generating runoff. The correlation between runoff yield and sediment yield was significantly positive (*p*<0.05) in all the plots, which confirmed that increasing runoff with larger energy was able to detach soil aggregation and cause severe erosion afterwards.

These results improved our understanding of litter as a protective layer and rainfall re-distributor. The data may be used to establish hydrological models to predict changes in runoff and soil erosion, and to provide scientific support for managing water resources in Northern China. Future studies should focus on the effects of slope, plot length, other litter types, and hydraulic characteristics (e.g., flow speed, erosive power, and shearing force) on runoff and soil erosion under litter cover to draw comprehensive conclusions.
